# Necrotizing pneumonia and sepsis due to Clostridium perfringens: a case report

**DOI:** 10.1186/1757-1626-2-50

**Published:** 2009-01-14

**Authors:** Cristina Palmacci, Manuela Antocicco, Lorenzo Bonomo, Fabio Maggi, Alberto Cocchi, Graziano Onder

**Affiliations:** 1 Department of Geriatrics, Policlinico A. Gemelli, Università Cattolica del Sacro Cuore, Rome, Italy; 2 Department of Bioimaging and Radiological Sciences, Policlinico A. Gemelli, Università Cattolica del Sacro Cuore, Rome, Italy

## Abstract

Clostridia are uncommon causes of pleuropulmonary infection. Clostridial species infecting the pleuropulmonary structures characteristically cause a necrotizing pneumonia with involvement of the pleura. Most cases have iatrogenic causes usually due to invasive procedures into the pleural cavity, such as thoracentesis or thoracotomy, or penetrating chest injuries. Rarely clostridia pleuropulmonary infections are not related to these factors. The clinical course of pleuropulmonary clostridial infections can be very variable, but they may be rapid and fatal. We report a rare case of necrotizing pneumonia and sepsis due to Clostridium perfringens not related to iatrogenic causes or injuries in an 82 years old woman.

## Background

Clostridia are uncommon causes of pleuropulmonary infection, especially in nonsurgical or nontraumatic patients [1]. Clostridial species infecting the pleuropulmonary structures characteristically cause a necrotizing pneumonia with involvement of the pleura. Most cases have iatrogenic causes: the Clostridia colonizing the skin by contamination may be introduced into the pleural space following invasive procedures, such as thoracentesis or thoracotomy, or penetrating chest injuries [1,2]. Other predisposing factors for necrotizing pneumonia are aspiration of oropharyngeal or gastric contents [1-4], pulmonary embolism with infarction (haematogenous seeding of infarcted lung tissue) [1]. In addition, this condition is often associated with chronic disease, such as diabetes or cirrhosis, and underlying pleuropulmonary pathology (pulmonary tuberculosis, chronic pleural effusions) [2,4]. Reported predisposing factors for systemic clostridial dissemination include intraoral pathology (carious teeth or gingival disease) and intrabdominal pathology such as malignancy and enteric vascular malformation or subdiaphragmatic infections [5,6]. The clinical course of pleuropulmonary clostridial infections can be very variable: clostridial pleuropulmonary infections are often indolent but they can be rapid and fatal [1].

## Clinical case

We present the case of a 82-years-old caucasian female, affected by paroxysmal atrial fibrillation (dual chamber pace-maker implanted in 2004), hypertension, normochromic normocytic anemia, arthritis, osteoporosis, cholelithiasis, admitted to our department for worsening of general conditions, hyperpyrexia and respiratory failure. At physical examination the patient was non-cooperative, drowsy and showed poorly productive cough and hyperpyrexia. Blood pressure was 100/70 mmHg, with a regular pulse rate of 90 bmp'. Blood gas analysis showed hypoxemia (paO2: 50.5 mmHg) and hypocapnia (pCO2: 33.1 mmHg). Chest examination showed abolition of vesicular murmur over the lower part of the left hemi-thorax and of the right lower lung fields. Abdominal examination showed presence of a voluminous right inguinal hernia. Laboratory examination documented neutrophilic leukocytosis and renal failure.

**Figure 1:**
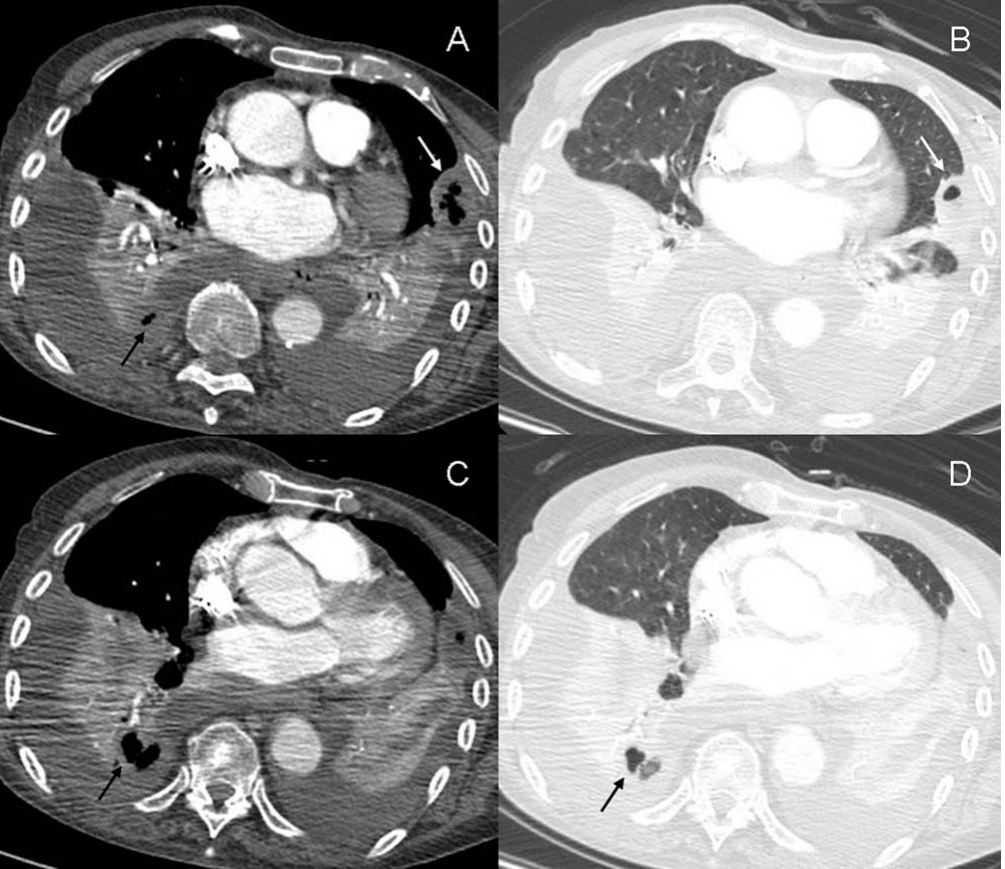
**Contrast-enhanced multi-slice CT of the thorax performed 48 hours after admission**. Multi-slice CT of the thorax confirmed presence of bilateral pleural effusion and consolidation of lower lobes and lingula; cavitation was revealed in the pleural-based consolidation of the lingula (Figure 2A-B, white arrows) and in the right lower lobe (Figure 2A-C-D, black arrows). These findings were considered indicative of a necrotizing, gangrenous pneumonia.

The chest X-ray showed aspecific findings of bilateral pleural effusion with consolidation of lower lobes and lingula. It was therefore undertaken oxygen therapy, hydrating, diuretic and empirical antibiotic therapy with cephalosporins and macrolides and blood cultures were performed. After 48 hours, a multislice CT scan of the chest was performed, which confirmed bilateral pleural effusion and consolidation of lower lobes and lingula; in right lower lobe and lingula areas of cavitation were also demonstrated, suggesting the hypothesis of a necrotizing, gangrenous pneumonia (Figure 1). An abdominal ultrasound did not show any relevant finding.

Clostridium perfringens was identified from two blood cultures and combined antibiotic treatment with Clindamicin and Amoxiciliin/Clavulanate was started. In the following weeks patient health conditions improved and a CT scan of the chest performed after two weeks of treatment showed marked reduction of pleural effusion and of extent of parenchymal consolidation with disappearance of cavitating areas. Antibiotic treatment was continued for two months. After this period, the patient fully recovered and she did not show any sign or symptom of pleuropulmonary disease.

## Discussion

Spontaneous pneumonia related to clostridium perfringens has rarely been described in the medical literature, while this condition seems more commonly associated with invasive procedures or penetrating chest injuries. Population based laboratory surveillance for invasive clostridium perfringens disease conducted in the population of the Calgary Health Region show an annual incidence of 0.83 per 100,000 with a striking age-related increased risk for acquisition [3]. This study shows that patients aged 65 years or older are at 12-fold higher risk for acquiring these infections as compared to younger patients [3].

Other risk factors for clostridium perfringens pneumonia are aspiration of oropharyngeal or gastric contents, pulmonary embolism with infarction (hematogenous seeding of infarcted lung tissue). In addition, this condition is often associated with chronic disease, such as diabetes or cirrhosis, and underlying pleuropulmonary pathology (pulmonary tuberculosis, chronic pleural effusions) is often found. Reported predisposing factors for systemic clostridial dissemination also include malignancies, in particular colon cancer. In this case, a endoscopy was not performed because the patient and her relatives refused the procedure.

## Consent

Written informed consent was obtained from the patient relative for publication of this case report and accompanying images. A copy of the written consent is available for review by the Editor-in-Chief of this journal.

## Competing interests

The authors declare that they have no competing interests.

## Authors' contributions

CP, MA, AC, FL and GO analyzed and interpreted the patient data regarding the infective disease. LB and FM performed and interpreted the chest X-ray and the CT scan. CP, MA and GO were responsible for writing the manuscript. All authors read and approved the final manuscript.
